# Supplementary figures and images for: Small Intestinal Contrast Ultrasonography (SICUS) in Crohn’s Disease: Systematic Review and Meta-Analysis
Source: J Clin Med. 2023 Dec 15;12(24):7714. doi: 10.3390/jcm12247714 (PMC10744114; doi:10.3390/jcm12247714)

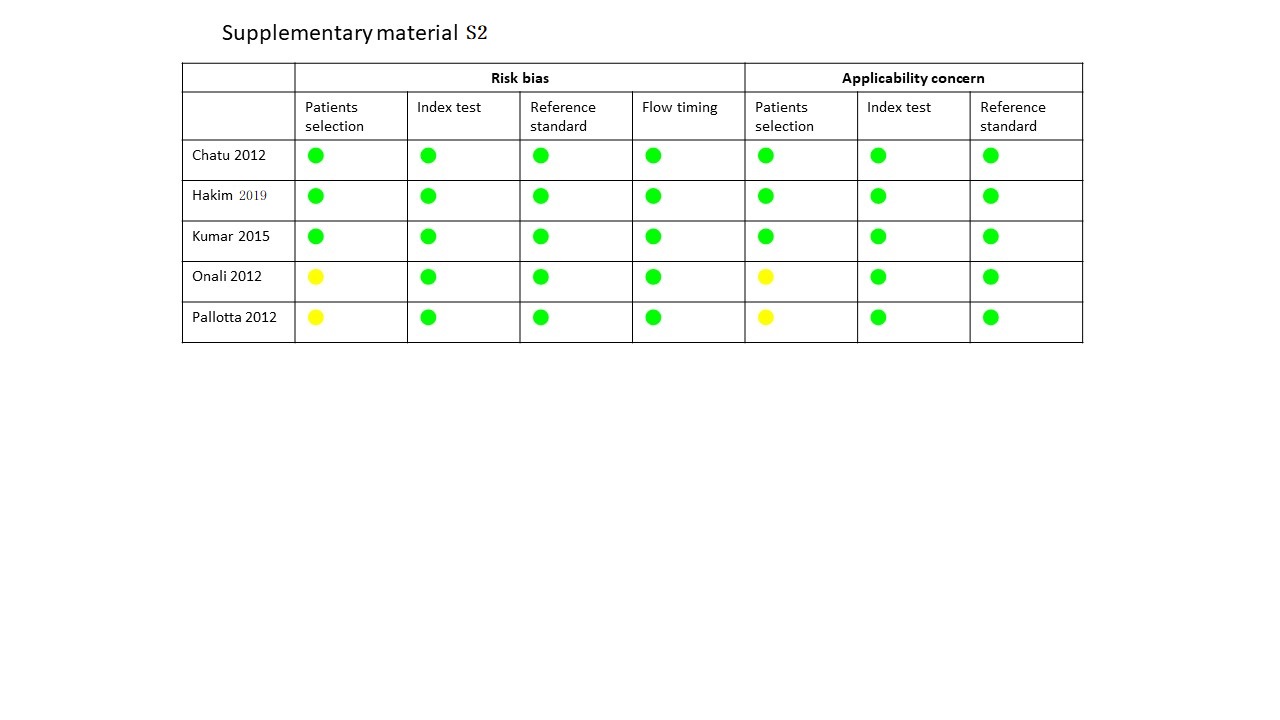

Supplement: Supplementary file 1 [file jcm-12-07714-s001.zip › Supplementary Material S2.jpg]
